# Supplementary material for: Music Therapy Assessment for Older Adults: Descriptive Mixed-Methods Study
Source: Behav Sci (Basel). 2024 Apr 23;14(5):354. doi: 10.3390/bs14050354 (PMC11118053; doi:10.3390/bs14050354)
Supplement: Supplementary file 1 [file behavsci-14-00354-s001.zip › Supplementary Material #S1 Music Therapy Assessment Older Adults MAOA.pdf]

Supplementary Material #1: Music Therapy Assessment Form (MAOA)

Background Information:

Client's Name: ____

Diagnosis: ____

Age: __________ Education: ______________________________

Ethnic Background: ____

Religion/Faith Practice:

____

Date of Informed Consent:

____

Consent Provided By: □ Client □ Substitute Decision Maker

Name of Person Providing

Consent:______________________________

□ Verbal □ Written Consent

Referred By:

____

Reason for

Referral:____

Assessment:

Cognitive

Orientation to Self: □ Yes □ No

Orientation to Surroundings (Date, Location, Season): □ Yes □ No

Ability to Read (Have client read: "I went for a walk with my dog by the lake.")

□ Some words □ Individual letters □ Full sentences □ Unable to read □ Not assessed

Ability to Write: □ Name □ Individual letters □ A sentence stating how they are feeling on day of assessment. □ Unable to write □ Not assessed

Attention Span (i.e. how long can the client focus on playing an instrument or engage in

conversation): □ Sustained (more than 3-5 minutes) □ Sporadic (Client is in and out of focus) □ Unable to attend

Ability to Follow Directions: □ Yes □ No

Comprehension: □ Understands basic concepts (fast/slow) □ Does not understand basic concepts

Ability to Choose: □ Not able □ Ability to choose between two items or instruments

Communication

Client is: □ Verbal □ Non-Verbal

Client has the following abilities: □ Vocalization □ Gestures □ Single words □ Phrases □ Sentences □ Eye contact

Psychosocial

Behaviour:

□ Pleasant □ Engaged □ Aggressive □ Perseverative □ Passive □ Self-stimulatory

□ Self-injurious □ Non-compliant □ Anxious □ Relaxed □ Withdrawn □ Involved

Ability to identify emotions:

□ In self □ In others □ Using language □ From choices (i.e pictures)

□ Unable to identify emotions

Ability to express emotions:

□ Using music □ Nonverbally □ Using language □ Using complex thought

□ Unable to express emotions

Self-Concept: □ Positive □ Negative □ Neutral

Inappropriate emotional expression: □ Denial □ Avoidant □ Not applicable

Affective Response

Beginning of session:

Affect: □ Flat □ Agitated □ Bright □ Other: ______

Attending behaviours: □ Asleep □ Eyes open □ Eye contact □ Active participation

End of session:

Affect: □ Flat □ Agitated □ Bright □ Other: ______

Attending behaviours: □ Asleep □ Eyes open □ Eye contact □ Active participation

Physical

Gross Motor: □ Ambulatory □ Non-ambulatory □ Movement with assistance

Fine Motor: □ Hold item independently □ Moves hand with intention □ Requires assistance

Coordination: □ Independent □ Supervised □ Support required

Hand dominance: □ Right □ Left □ Ambidextrous

Sensory limitations: □ Vision □ Hearing

Musical

Sings: □ All words and phrases □ Half □ Less than half □ Vocalizes/makes sounds

Vocal ability and timbre: □ Dynamics □ Pitch accuracy □ Range □ No vocal range

Explores percussion instruments presented: □ Yes □ No

Imitates rhythm: □ Simple rhythms □ Complex rhythms

Summary

Cognitive:

Communication:

Psychosocial:

Affective/Emotional

Physical:

Musical:

Initial Goals

□ Provide sensory stimulation

□ Increase social interaction/decrease isolation

□ Increase motivation

□ Increase self-esteem/self-concept

□ Maintain/improve orientation

□ Maintain/improve physical well-being

□ Provide outlet for emotional expression

□ Decrease anxiety/agitation

□ Increase relaxation

□ Provide a creative-expressive outlet

□ Manage dysfunctional behaviours:

    □ Enhance mood, reduce feelings of depression

    □ Connect with identity

    □ Spiritual support

□ Pain control

□ Other

Recommended Services

□ Individual Music Therapy □ Group Music Therapy □Not Suitable for Music Therapy

□ Individual Music Psychotherapy (Additional assessment to follow prior to final recommendation) □ Recreational Music Opportunities

Music Therapist Signature: ___________________ **Date:**

Created by: XX
