# Supplementary material for: Music Therapy Assessment for Older Adults: Descriptive Mixed-Methods Study
Source: Behav Sci (Basel). 2024 Apr 23;14(5):354. doi: 10.3390/bs14050354 (PMC11118053; doi:10.3390/bs14050354)
Supplement: Supplementary file 1 [file behavsci-14-00354-s001.zip › Supplementary Material #S2 Music Therapy Assessment Older Adults MTAOA 2(1).pdf]

**Supplementary Material #2: Music Therapy Assessment Form Revised (MTAOA-R)** (highlighted text shows what was changed from the original MTAOA)

## Background Information:

Client/Service User/Consumer's Name (C): ______________________________

Pronouns: ______________________ Gender: ____________________

Date(s) of Assessment:_____________________________________________

Diagnosis: _____________________________________________________

Medications: ____________________________________________

Date of Birth: ______________ Age: _____________

Education: _______________________________________

Cultural Information: ____________________________________________

Religion/Faith Practice: _________________________________________

Date of Informed Consent: _________________________________________

Consent Provided By: □ Client/Service User/Consumer □ Substitute Decision Maker □ Facility

Name of Person Providing Consent:___________________________________

Check all that Apply: □ Verbal Consent □ Written Consent

Referred By: __________________________________________________

Reason for Referral: ______________________________________________

Has (C) participated in music therapy in the past? □ Yes □ No

If yes, please provide dates if known, reasons and/or format-individual/group etc.

_______________________________________________________________________________

## Assessment:

### Cognitive

Orientation to Self: □ Yes □ No

Orientation to Surroundings (Date, Location, Season): □ Yes □ No

Using the accompanying card with this statement have (C) read: "I went for a walk with my dog by the lake." Indicate the response.

□ Some words □ Individual letters □ Full sentences □ Unable to read □ Not assessed

Writing: □ Name □ Individual letters □ A sentence stating how they are feeling on day of assessment. □ Unable to write □ Not assessed

Attention Span (i.e. how long can (C) focus on playing an instrument or engage in conversation):

□ Sustained (3-5 minutes) □ Sporadic (Client is in and out of focus) □ Unable to attend

□ Other noting specific time or contributing factors ________________________________

Following Directions:

□ 1-step □ 2-step □ 3+ steps □ Unable to follow directions

Comprehension:

□ Understands basic concepts (fast/slow) □ Does not understand basic concepts

Making Choices:

□ Not able □ (C) chose between two items or instruments

Standardized Test Scores and Dates if Applicable i.e. MOCA:

______________________________

**Communication**

(C) is: □ Verbal □ Non-Verbal

(C) uses the following to communicate: □ Vocalization □ Gestures □ Single words □ Phrases □ Sentences □ Eye contact □ Symbols □ Communication device

Intelligibility of Speech: □ 100% of meaning conveyed □ 50% of meaning conveyed □ Other ___________

Articulation □ Clear □ Parts of Speech are clear □ Stuttering present

Vocal Volume □ (C) expresses no concern over volume of voice □ (C) expresses a desire to increase volume □ Volume is soft without support

Presence of Aphasia: □ Receptive □ Expressive

**Psychosocial**

Behaviours:

History of □ Anxiety □ Aggression towards others □ Depression □ Perseverative Behaviour □ Other ______________________

During Assessment (C) displayed or noted □ Anxiety □ Aggression towards others □ Low mood □ Perseverative Behaviour

□ Self-injurious behaviour □ Other ______________________

Identifying emotions:

□ In self □ In others □ Using language □ From choices (i.e pictures)

□ Unable to identify emotions

Expressing emotions:

□ Using music □ Nonverbally □ Using language □ Using complex thought

□ Unable to express emotions

History of Trauma

____________________________________________________________

Self-Concept:

□ Positive □ Negative □ Neutral □ Not assessed

**Affective Response**

Beginning of session:

Affect: □ Flat □ Agitated □ Bright □ Other: ______

Attending behaviours: □ Asleep □ Eyes open □ Eye contact □ Active participation

End of session:

Affect: □ Flat □ Agitated □ Bright □ Other: ______

Attending behaviours: □ Asleep □ Eyes open □ Eye contact □ Active participation

**Physical**

Gross Motor:

□ Ambulatory □ Non-ambulatory □ Movement with assistance □ Movement with supervision

Mobility Devices (Select all the apply) □ Cane □ Walker □ Wheelchair □ Bed bound

Fine Motor:

□ Hold item independently □ Moves hand with intention □ Requires assistance

Coordination:

□ Independent □ Supervised □ Support required

□ Can cross the midline

□ Balance □ History of Falls if known ________________________________

Hand dominance:

□ Right □ Left □ Ambidextrous

Sensory:

Vision □ (C) uses glasses or other assistive device □ (C) notes challenges i.e. ________________□ Difficult to assess □ Did not appear to see some items

Hearing: □ (C) states they can hear □ appears to hear verbal and music exchanges □ Difficult to assess □ used assistive device to hear

**Musical**

Sings:

□ All words and phrases □ Half □ Less than half □ Vocalizes/makes sounds

Vocal ability and timbre:

□ Dynamics □ Pitch accuracy □ Range □ No vocal range

Explores percussion instruments presented:

□ Yes □ No

Imitates rhythm:

□ Simple rhythms □ Complex rhythms

Music Preferences/
Styles/Artists____________________________________________________________

**Summary**

Cognitive:

Communication:

Psychosocial:

Affective/Emotional

Physical:

Musical:

**Initial Goals**

□ Provide sensory stimulation

□ Increase social interaction/decrease isolation

□ Increase motivation

□ Provide cognitive stimulation

□ Increase self-esteem/self-concept

□ Maintain/improve orientation

□ Maintain/improve physical well-being

□ Provide outlet for emotional expression

□ Decrease anxiety/agitation

□ Increase relaxation

□ Provide a creative-expressive outlet

□ Manage responsive behaviours i.e. wandering

□ Enhance mood, reduce feelings of depression

□ Connect with identity

□ Pain control

□ Other

**Recommended Services**

□ Individual Music Therapy □ Group Music Therapy □Not Suitable for Music Therapy

□ Individual Music Psychotherapy (Additional consent form to be signed)

□ Recreational Music Opportunities □ Music Listening (Set up with Playlist)

□ Singing Group (Choir) □ Music Program with Caregiver

**Music Therapist Signature**: ________________________________ Date: _______________

Revised by: XX
