# Supplementary material for: Music Therapy Assessment for Older Adults: Descriptive Mixed-Methods Study
Source: Behav Sci (Basel). 2024 Apr 23;14(5):354. doi: 10.3390/bs14050354 (PMC11118053; doi:10.3390/bs14050354)
Supplement: Supplementary file 1 [file behavsci-14-00354-s001.zip › Supplementary Material #S3 Assessment Research Study Interview Questions.pdf]

Supplementary Material #3 : Assessment Research Study Interview Questions

1. Describe and/or discuss your experience using the MTAOA?
2. Describe the client/consumer/service user with whom you used the MTAOA form?
3. Of these populations, which did you find the MTAOA most useful for, or conversely was there a population which you felt it was particularly unsuited?
4. Do you think the MTAOA can work with other client/service user/consumers? If so who?
5. How many assessments did you administer before taking the research study survey?
6. Did you suggest adding or removing any items from the MTAOA? If so which ones and why?
7. Are you continuing to use the MTAOA or will you continue to use the MTAOA as part of your music therapy practice? Why or why not?
8. How did you use the MTAOA? Eg: did you fill it out during the initial session or after the initial session? Or did you use it over a few sessions?
9. What parts of the MTAOA did you find most useful and needed and why?

10. What parts of the MTAOA did you find least useful or were not needed and why?
11. Was any of the terminology unclear, vague, unhelpful, or does not tell us anything important? If so, which terminology and what terminology would you use instead?
12. Are there any goals you would remove from the initial goal checklist and/or add that are more relevant to your client/consumer/service user or practice? If so, what would you add or remove and why?
13. Would you prefer to have space to list preferred music under the music section or as part of the initial background information?
14. Is there anything you would change to make this assessment more useful in your practice? If so, what would you change?
15. Do you think there is a benefit of implementing a standardized versus non-standardized assessment in practice? Why or why not?
16. Is there anything that was not addressed in the survey or interview that you would like to share?
